# “Computational analysis on verbal fluency reveals heterogeneity in subjective language interests and brain structure”

**DOI:** 10.1016/j.ynirp.2023.100159

**Published:** 2023-02-19

**Authors:** Francilia Zengaffinen, Antje Stahnke, Stephan Furger, Roland Wiest, Thomas Dierks, Werner Strik, Yosuke Morishima

**Affiliations:** a Translational Research Center, University Hospital of Psychiatry, University of Bern, Bern, Switzerland; b University Institute of Diagnostic and Interventional Neuroradiology, Inselspital, University of Bern, Bern, Switzerland

**Keywords:** Language, SyNoPsis, Computational analysis, LSA, VBM, Healthy cohort, Psychosis

## Abstract

Language is an essential higher cognitive function in humans and is often affected by psychiatric and neurological disorders. Objective measures like the verbal fluency test are often used to determine language dysfunction. Recent applications of computational approaches broaden insights into language-related functions. In addition, individuals diagnosed with a psychiatric or neurological disorder also often report subjective difficulties in language-related functions. Therefore, we investigated the association between objective and subjective measures of language functioning, on the one hand, and inter-individual structural variations in language-related brain areas, on the other hand.

We performed a Latent Semantic analysis (LSA) on a semantic verbal fluency task in 101 healthy adult participants. To investigate if these objective measures are associated with a subjective one, we examined assessed subjective natural tendency of interest in language-related activity with a study-specific questionnaire. Lastly, a voxel-based brain morphometry (VBM) was conducted to reveal associations between objective (LSA) measures and structural changes in language-related brain areas.

We found a positive correlation between the LSA measure cosine similarity and the subjective interest in language. Furthermore, we found that higher cosine similarity corresponds to higher gray matter volume in the right cerebellum. The results suggest that people with higher interests in language access semantic knowledge in a more organized way exhibited by higher cosine similarity and have larger gray matter volume in the right cerebellum, when compared to people with lower interests.

In conclusion, we demonstrate that there is inter-individual diverseness of accessing the semantic knowledge space and that it is associated with subjective language interests as well as structural differences in the right cerebellum.

## Nomenclature

LSA =: Latent Semantic Analysis
VFT =: Verbal Fluency Task
SAM-Q =: Speech- Affect- Motor function Questionnaire

## 1 Introduction

Language is an essential instrument to conduct complex communication, knowledge transfer, and abstract thought in humans (Berwick et al., 2013; Locke and Bogin, 2006). There is huge inter-individual heterogeneity in language functions: Some are good in communication and others are good in abstract thought. Those functions are often impaired in psychiatric and neurological disorders (De Boer et al., 2018).

A quick assessment of language function often used in clinical settings (e.g., psychiatry, neurology) is the verbal fluency test (VFT). It is often used since it involves semantic memory, context processing and controlled retrieval, and gives a quick overview of an individual's general language functioning (Docherty et al., 2011). The VFT can be used in two ways, either as phonological or as a semantic test, in which a participant is asked to generate as many items as possible of a set in a predetermined amount of time. When the phonological VFT is used, a participant is given a letter of the alphabet (e.g., F, S, A) and is asked to produce as many words as possible starting with this letter. In the semantic VFT, a participant is asked to produce as many items as possible of a certain category (e.g., fruits) within the time constraint, and the VFT provides predictors for poor language functioning (Lezak et al., 2012).

For example, a decrease in the number of items produced during the VFT is one indicator of language abnormalities (Docherty et al., 2011). Other classical measures, such as number of errors and repetitions, also provide insights into language functioning.

In the past decade, various computational approaches have been applied to study human language functions in healthy and clinical cohorts. The Latent semantic analysis (LSA) gained a lot of interest in psychiatric research, especially its application to the VFT, since this method can deliver objective descriptions of language abnormalities as an automatic measurement of discourse coherence, syntactic complexity, poverty of content, referential coherence, and metaphorical language, and therefore reveal a picture of language anomalies in different clinical cohorts (Corcoran and Cecchi, 2020).

LSA is a statistical computation method, which is applied to a large corpus of text to extract the contextual usage of word meaning (Landauer et al., 1998). The underlying idea is that the collective of all the possible contexts a word can or cannot appear in provides a measure that determines the similarity of meanings of words or set of words to each other (Landauer et al., 1998). Each word is represented as a vector in the high-dimensional semantic space (Foltz et al., 1998). The vector length is one of the measures calculated by the LSA and represents the information value of a word, meaning the word “unusualness”. High frequency or “usual” words carry less information value than low frequency or unusual words and therefore high frequency words produce smaller vectors lengths. Another important measure is the average cosine similarity or average coherence, which is the average of all cosine values between each word and the word that immediately follows it (Elvevag et al., 2007; Foltz et al., 1998). This measure represents how we retrieve answers from the semantic space. A higher average cosine similarity reflects more sequential retrieval of semantically similar words, while the lower value reflects more random retrieval of semantically distant words. Studies with the LSA analysis showed reasonable sensitivity and specificity in groups of individuals with a specific condition, such as psychosis, dementia, Alzheimer's disease, Parkinson's disease, autism spectrum disorder and ADHD, as compared to healthy controls and healthy controls compared to a human manual rating (De Boer et al., 2018). Also, LSA is a more reliable and in-depth method than the human counterpart of diagnosticians using rating of symptoms as a method to address language dysfunction.

As mentioned above, studies often examine only the objective measures of language dysfunction. However, psychiatric and neurological patients with language dysfunction often report in clinical interviews that they subjectively feel difficulties in their language-related abilities, such as reading, writing, thinking, speaking and communication. In the current study we investigated the association between self-reported interests in language-related activities and text coherence during VFT, measured with the LSA. Therefore, we focused also on the subjective report of language interest, instead of only on the objective performance. Furthermore, we were interested in the association between subjective and objective measures of language-related functions and neurobiological mechanisms in the brain, specifically using voxel-based brain morphometry (VBM). Previous studies reported that not only cortical areas including the inferior and middle frontal gyrus, fusiform gyrus, and inferior, middle and superior temporal gyrus, but also subcortical structures, such as the cerebellum and basal ganglia are affected in neurological patients (Pereira et al., 2009; Rodríguez-Aranda et al., 2016; Silveri, 2021).

In the current study, we were interested if there is a difference in strategy of verbal production between individuals with a high subjective interest in language compared to those who have a less subjective interest. To this end, we applied the LSA to the semantic VFT and investigated the association with subjective interest in language. We also performed VBM analysis to test for the association of the structural correlates with subjective and objective measures of language.

## 2 Material and methods

### 2.1 Participants

This study is a part of an extensive project on three domains affected in psychosis “language, affect and motor function” in a healthy cohort (Furger et al., 2020). One hundred twenty-four healthy participants were included. They were recruited through the University of Bern, high schools and colleges in Bern, as well as at special events (e.g., the annual museum's night). Each participant underwent a thorough screening process which included the following exclusion criteria: any current mental health disorder or a history of a psychotic disorder (lifetime), head injury with loss of consciousness, contraindication for MRI (e.g., claustrophobia, pregnancy, metal implants), learning disability (e.g., dyslexia) or substance abuse disorder (except tobacco). Participants were between 16 and 60 years of age and were fluent in German. Twenty-three participants were excluded from the analysis because of missing VFT task data, MRI data, or being over the age of 40. In this study we have excluded participants over age of 40, because gray matter volume is markedly reduced after age of 40, and this would predominate other inter-individual variances. Additional VBM analysis was performed with a subcohort of age between 21 and 40 (see supplement). Each participant gave their written informed consent before participation. The study protocol was approved by the local ethic committee of the Canton Bern (Kantonale Ethikkommission Bern, approval number 2016–01261) and is in accordance with the Declaration of Helsinki (World Medical, 2001).

### 2.2 Speech-Affect-Motor function questionnaire

The SAM-Q (Speech-Affect-Motor function questionnaire) is a study-specific questionnaire which was used to inquire the subjective interests in three cognitive domains (language, affect and motor function). This questionnaire is an extension of the Bern Psychopathology Scale, a three-dimensional assessment of pathological symptoms in psychotic disorders (Strik et al., 2017; Strik et al., 2010). In the Bern Psychopathology Scale, each question has to be answered on a seven-point Likert scale from −3 to +3, about their self-perceived changes in behavior and evaluation by examiners in these domains. The negative rating score reflects an inhibition of a represented behavior (i.e. mutism), whereas a positive score reflects a disinhibition of the behavior (i.e. pressured speech) respectively. A rating at zero would be a nonexistence of the represented pathological behavior. Correspondingly, in the SAM-Q questionnaire a positive rating would reflect great interests in a domain and a negative rating would represent no or low interest.

In this part of the study, we assessed/focused on the language domain. Participants had to answer the following question: “Ich bin ein sprachlicher Mensch (I am a person, who is apt in language)”.

### 2.3 Verbal fluency task

Each participant was asked to name as many animals as possible in a minute. Participants were instructed not to name category names (e.g., Mammals, Primates etc.) or breeds of animals (e.g., Labrador, Shepard etc.).

Before performing the animal VFT task, each participant was familiarized with the task with the following instruction: “As soon as I say “start”, please name as many flowers as possible, like rose, tulip, gardenia, in the next 60 s that come to mind. There are two rules that you must follow. Category names like “garden flowers” or breeds like “wild roses, tea roses” are not allowed and will be counted as errors.” Once we confirmed the participants understood the instruction, the examiner started the test.

The category tested was animals and the answers were recorded by an audio recorder, and the answered word sequence was manually transcribed into an excel file. The file included the raw data (animal names), the score number, which was the total number of correctly named items. Items which were either incorrect or named twice were not added to the score but were counted as errors.

### 2.4 Latent semantic analysis

We used the “LSAfun” package implemented in R software (https://www.r-project.org/) for LSA analysis of the VFT data. Pretrained semantic spaces are provided by the developer of the package, and we used “de_wiki” pre-trained model (https://sites.google.com/site/fritzgntr/software-resources/semantic_spaces). The model includes 526,004 words with 400 dimensions of word vectors.

For each participant, we first checked whether each word of the answered word sequence was included in the word vector model. If a particular word is not included in the model, we assigned ‘NA’ in the word sequence. As the VFT does not allow duplication of words in the answered word sequence, a word that appeared more than once was coded as ‘NA’. Number of correct words was counted. For each word, we calculated the length of a word vector and averaged across all words except ‘NA’.

Then, we calculated the cosine similarity between adjacent two words. If one or two of two adjacent words was ‘NA’, we omitted the calculation of cosine similarity and assigned ‘NA’. Finally, we averaged the cosine similarity values excluding ‘NA’.

### 2.5 Voxel-based brain morphometry

We performed voxel-based morphometry (VBM) (Ashburner and Friston, 2000) to assess the association of inter-individual variability in gray matter volume with the performance of the VFT.

We used a 3T Prisma MRI scanner (Siemens, Germany) at the Institute of Diagnostic and Interventional Neuroradiology, Inselspital, University of Bern. High-resolution T1-weighted structural images were obtained with a 3D magnetization-prepared rapid acquisition with a gradient echo (MPRAGE) sequence (TR = 2300 ms, TE = 2.98 ms, flip angle = 9 deg, Field of view = 256 mm × 256 mm, 160 sagittal slices, voxel size = 1 mm isotropic) with 11 min total acquisition time.

We used SPM12 (http://www.fil.ion.ucl.ac.uk/spm) and CAT12 toolbox (http://www.neuro.uni-jena.de/cat/) in MATLAB 2018a (Mathworks, Inc, Natwick, MA, USA) for T1 image preprocessing and SPM analysis. First, using the CAT 12 toolbox, T1 images were segmented into gray matter (GM), white matter (WM), and cerebral spinal fluid (CSF) in native space and the GM image was normalized into MNI space and resampled to 1.5 mm isotropic voxels. Normalized GM images were smoothed with a Gaussian kernel of 6 mm full width half maximum (FWHM).

The preprocessed GM images were entered into a multiple regression model in SPM12. In this model, the gray matter density variance was explained with the factors of the VFT results (mean cosine similarity), SAMQ language score, number of correct words in the VFT and the demographic variables (age and gender).

To deal with differences in brain size across participants, we obtained total intracranial volume (ICV) estimated by the CAT12 toolbox. We included the ICV values as a covariate of no interest in the regression model. We set the significance threshold for the exploratory analysis at p < 0.05 family-wise error corrected at a voxel level.

### 2.6 Statistical analysis

We performed correlation and multiple regression with SPSS software (version 27, IBM Corp, Armonk, NY, USA). We considered p-value less than 0.05 as significant.

## 3 Results

### 3.1 Cohort description

**Fig. 1:**
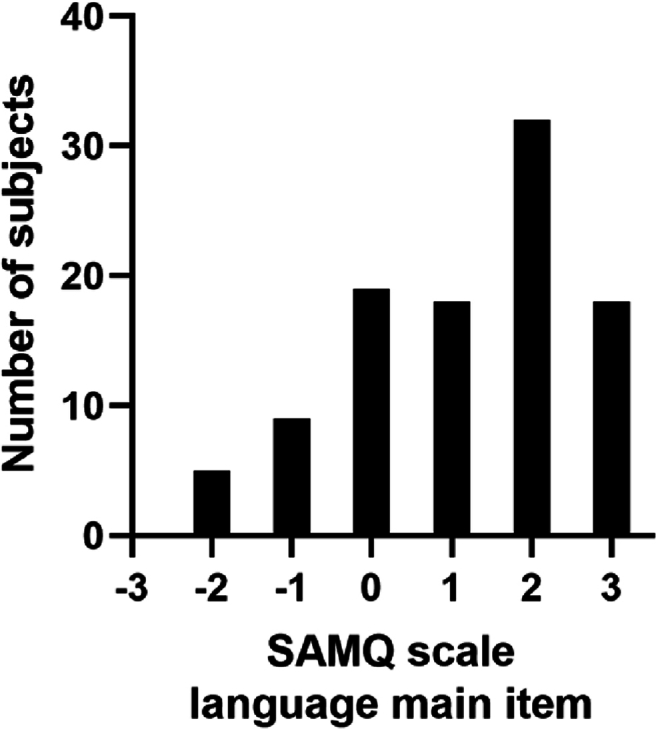
Distribution of the ratings of each participant on their perceived language interest. Negative scores correspond to low or no interest in language, whereas positive scores correspond to high interest in language.

Demographic data of the participants included in the analysis is listed in Table 1. The distribution of the SAMQ language item is depicted in Fig. 1. Approximately two thirds of the participants rated themselves as having a high interest in language.

**Table 1:** Demographic data of total participants (n = 101).

Gender (f/m)	78/23
Age (years, SD, min/max)	24.34 (5.34, 16/40)
Handedness EHI (right/left/mixed)	84/8/9
SAMQ Language main item (number of participants) (rating scale number)	5/9/19/18/32/18 (−2/−1/0/+1/+2/+3)

Abbr.: SAMQ = Speech-Affect-Motor function Questionnaire, EHI = Edinburgh Handedness Inventory.

### 3.2 Descriptive statistics of LSA results

The average number of correct items during the VFT was 25.62. Mean values of cosine similarity and vector length across participants were 0.364 and 2.811, respectively, and we observed considerable inter-individual differences (see Table 2).

**Table 2:** Descriptive data of LSA analysis (n = 101).

	Minimum	Maximum	Mean	Standard deviation
Number of Words	14	41	25.62	5.479
Cosine Similarity	0.288	0.422	0.364	0.025
Vector Length (a.u.)	2.514	2.990	2.811	0.098

### 3.3 Multiple regression

To examine whether the subjective interest in language has any impact on how they access their semantic knowledge, we performed multiple regression analysis. We used mean cosine similarity as a dependent variable and included SAMQ language score, number of correct items, gender, and age as independent variables. We found that the beta estimate for the SAMQ language score is significantly correlated with mean cosine similarity (p = 0.018) (Table 3). In contrast, mean vector length was not correlated with the SAMQ language score, but significantly negatively correlated with number of items answered (p < 0.001) (Table 4).

**Table 3:** Multiple regression with mean cosine similarity as dependent variable.

	Beta estimate	Std. Error	t-ratio	prob.
Independent Variables				
Number of Items	0.000	0.000	0.830	0.408
SAMQ language main Item	0.004	0.002	2.417	0.018*
Age	−0.001	0.000	−1.342	0.183
Gender	−0.005	0.006	−0.857	0.394

Constant	0.374	0.022	16.865	<0.001*

Legend: all results are rounded up to three decimals and depicted with an * if they are significant (p < 0.05).

**Table 4:** Multiple regression with mean vector length as dependent variable.

	Beta estimate	Std. Error	t-ratio	prob.
Independent Variables				
Number of Items	−0.007	0.002	−4.282	<0.001*
SAMQ language main Item	−0.007	0.006	−1.118	0.266
Age	−0.001	0.002	−0.499	0.619
Gender	−0.012	0.022	−0.558	0.578

Constant	3.047	0.081	37.714	<0.001*

Legend: all results are rounded up to three decimals and depicted with an * if they are significant (p < 0.05).

### 3.4 VBM

**Fig. 2:**
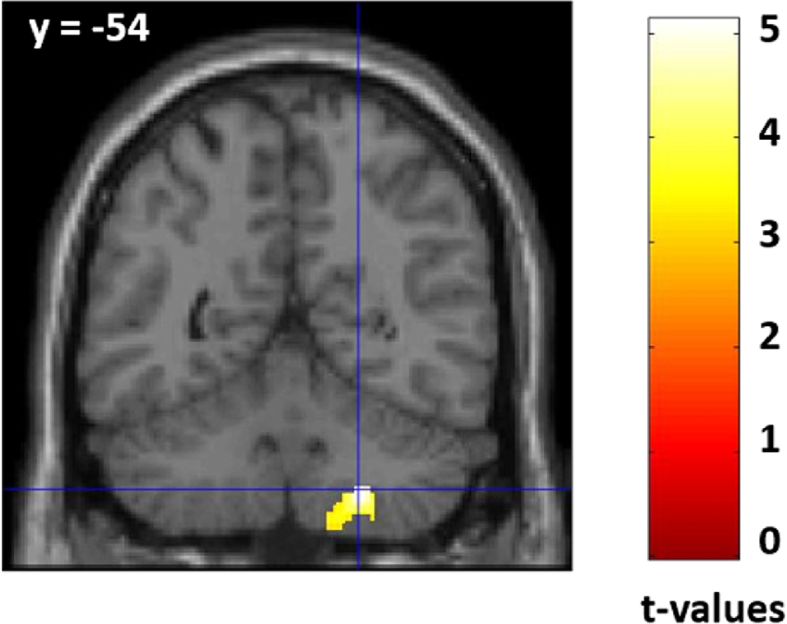
Statistical parametric map for correlation between LSA cosine similarity and gray matter volume. Right cerebellum was significantly correlated with LSA cosine similarity (peak: [x, y, z] = [22.5, −54, −46.5]; t-value = 5.11; p = 0.034, voxel-wise whole-brain FWE corrected for multiple comparison). For visualization purposes, voxels that survive at p < 0.001 uncorrected (t = 3.16, cluster size = 506) are depicted.

We further examined whether inter-individual variation in brain structure can account for mean cosine similarity, the heterogeneity of access to the semantic knowledge. To this end, we performed VBM analysis and found that the gray matter volume in the right cerebellum was highly correlated with mean cosine similarity (peak coordinate [x y z] = [22.5, −54, −46.5], t = 5.11, p (voxel-wise FWE) = 0.034, cluster size = 10) (Fig. 2). The significant cluster corresponds to the subregion hemispheric VIIIb (Diedrichsen et al., 2009; Schmahmann et al., 1999). Higher gray matter volume corresponded to higher mean cosine similarity. However, we did not find any significant correlation of a GM volume with subjective interest in language and number of correct words in whole brain. Furthermore, we performed correlation analysis between the right cerebellum ROI and subjective interest in language and number of correct words. We found no significant correlation between GM volume in the right cerebellum and subjective interest in language (r (100) = −0.03, p = 0.80) and between GM volume in the right cerebellum and number of correct words (r (100) = −0.06, p = 0.56).

We also found gray matter volume was significantly negatively correlated with age in variety of brain areas, but not in the cerebellum (Supplementary Fig. 1).

As gray matter thinning continues until the age of 20, we further performed another VBM analysis including only subjects between 21 and 40 years of age. We found brain areas showing age-dependent decrease in GM volume was markedly reduced (Supplementary Fig. 2). We again confirmed that GM volume in the right cerebellum was significantly correlated with mean cosine similarity (peak coordinate [x y z] = [21, −52.5, −48], t = 4.62, p (cluster-wise FWE) = 0.01, thresholded at p < 0.001 uncorrected, cluster size = 605, Supplementary Fig. 3).

## 4 Discussion

In the current study, we investigated the link between a subjective interest in language and objective language task performance, and neurobiological correlates of inter-individual heterogeneity of accessing semantic knowledge.

We performed LSA analysis on VFT and found positive correlation between cosine similarity during the VFT and subjective interest in language. Using VBM, we also found the cosine similarity during the VFT was positively correlated with gray matter volume in the right cerebellum.

The advantage to use the LSA is that we can obtain more detailed information of the text data in a quantitative way. This can be categorized into two domains. One is information of each answered word, obtained by vector length. Shorter vector length represents more common words, while longer vector length reflects less frequent words. The other is information between answered words. Cosine similarity is a measure of the similarity between two-word vectors, and a higher value indicates two words are semantically close to each other, while lower value indicates semantically distant.

Previous studies applied the LSA methods to clinical cohorts included psychosis, autistic spectrum disorders, dementia including mild cognitive impairments (see for an overview De Boer et al. (2018)). Various types of text data (text, sentences, or words) were used from storytelling, narrative interview, prose recall to semantic and phonological verbal fluency tasks. Most of the psychosis and dementia studies showed a decrease in number of the produced correct items. A study revealed that the LSA coherence score significantly differed between high and low severity of thought disturbance in individuals with schizophrenia (Elvevag et al., 2007). In addition, a significant difference was found between individuals with schizophrenia and healthy controls. In the same study, a schizophrenia subgroup with high levels of clinical formal thought disorder (FTD) showed a lower coherence score in comparison to the group with low level of FTD and in comparison, to healthy controls. Interestingly, there was no significant difference of the coherence score between the low level FTD group and the healthy controls. A previous study by Lundin et al. (2022) found that individuals with early psychosis showed lower mean cosine similarity than matched healthy controls. A study with individuals diagnosed with Alzheimer (AD) and Mild Cognitive Impairment (MCI) revealed that AD group produces higher similarity and relatedness scores when compared to MCI group, and the AD group answered less correct items than MCI group. This unexpected result of higher scores in similarity and relatedness could be due to the individuals with AD ability to produce only highly semantically related words with limited semantic space (Pakhomov et al., 2012). Hoffman et al. (2014) showed that individuals with semantic dementia were more likely to produce more highly frequent and abstract words during an autobiographical interview than compared to the healthy control, who produced more low frequency and high-imageability words. Important to note is that the higher frequency words were of a broader category (place, thing), instead of the more semantic low diversity words (garage, spanner). Another study showed that individuals with high functioning autism produced similar semantic content when narrating from a picture book compared to healthy controls. However, in a narrative recall task they showed diminished semantic quality when compared to the healthy controls (Losh and Gordon, 2014). In sum, previous research revealed that LSA approaches can detect various types of functional impairments of language in clinical populations. In addition, it has been revealed that the number of items is associated with the broadness of an individual semantic space, while semantic similarity is associated with the strategy to explore the semantic space. Our study has revealed that the strategy to explore the semantic space is associated with subjective interests in language as well as gray matter volume in the right cerebellum.

In the current study, we found that positive correlation between subjective interest of language and mean cosine similarity value. Higher value of cosine similarity indicates two words are semantically closer to each other. Therefore, we could interpret people with a high interest in language access and retrieve their knowledge in a more organized way, while people with lower interest of language tend to be less organized. However, there is another possible interpretation. Semantic spaces used for the LSA analysis are trained by the Wikipedia or news articles. Higher cosine similarity reflects two words are just close to each other in the semantic space, and lower value of cosine similarity are just distant. The semantic knowledge of people with lower mean cosine similarity may be just different from the semantic space derived from training data sets. We also found no significant association between mean cosine similarity and number of correct items, suggesting that organized access to semantic knowledge may be independent from retrieval of knowledge.

In fact, a study focused on an ADHD revealed that people with ADHD showed more creativity as well as lower semantic coherence during the word association task (White and Shah, 2016) and that verbal fluency is only marginally decreased, while subjective perception of their cognitive functions are more impaired (Fuermaier et al., 2015). Therefore, we argue that the lower value of semantic similarity measures without decrease of overall performance (i.e. number of correct items) itself does not indicate the lower ability in semantic knowledge or language, but the difference in strategy to access in semantic knowledge.

As mentioned in paragraph 2.2 Speech-Affect-Motor function questionnaire, three cognitive domains are associated with psychopathology of psychiatric disorders and in the language domain it assesses broad spectrum of language-related functions including subjective rating by patients (Cavelti et al., 2018; Kircher et al., 2014; Krueger et al., 2005; Strik et al., 2010; Viher et al., 2018). In the current study, we collected subjective ratings of interests in language-related activities only from a healthy cohort. In future research we would like to investigate the relationship between subjective ratings and objective neuropsychological assessment in language related functions in broader spectrum of healthy and patient cohorts.

In the current study, we also examined the association of vector length. We did not find a significant association between vector length and subjective interest of language, but we only found that mean vector length was significantly negatively correlated with number of correct items. Although that negative correlation is consistent with a previous study (Lundin et al., 2022), which is counterintuitive to name more unusual animals, resulting in longer mean vector length. However, the actual data suggests that people who produced more correct items answered more popular animals, compared to people who produced less correct items. Another study showed similar results with older individuals with schizophrenia (Holshausen et al., 2014). The coherence was not correlated with performance in the verbal fluency task. However, the semantic fluency was associated with average vector length. Similar to the study mentioned before, a negative correlation was found, which could suggest that individuals with retrieval difficulties would not generate usual responses, meaning fewer correct responses and less high frequencies words. Another interesting result occurs in a longitudinal study with a healthy elderly cohort, where LSA was applied to the semantic verbal fluency task and the cohort was followed for 13 years. The study demonstrated that people answered more semantically related items in the semantic verbal fluency task had lower risk to develop to dementia 13 years later (Pakhomov and Hemmy, 2014).

Using VBM, we found the positive correlation between mean cosine similarity and gray matter volume in the right cerebellum, while we did not find any association of gray matter volume with number of correct items in the VFT or subjective rating of interest in language. The results suggest that people who have larger gray matter volume in the right cerebellum access their semantic knowledge space in a more organized manner. The activation of the cerebellum in language tasks, even after controlling for motor aspects, is a robust finding. Studies in neuroanatomy, clinical settings and fMRI repeatedly found the involvement of the cerebellum in language task without motor responses (Marien and Borgatti, 2018; Murdoch, 2010). Neuroanatomy studies found contralateral reciprocal connections between the right cerebellum and the left frontal language dominant areas (Leiner et al., 1987; Leiner et al., 1989, 1991, 1993). Clinical studies further strengthen the involvement of the cerebellum in language processes. A meta-analysis of studies with individuals with focal cerebellar lesions showed that these individuals perform slightly worse in semantic and phonological verbal fluency task when compared to healthy controls (Ahmadian et al., 2019). Another study from individuals with non-demented Parkinson (Pereira et al., 2009) showed that not only left temporal and frontal gray matter density correlated with semantic fluency scores, but also bilateral cerebellar areas as well. FMRI studies also show involvement of the cerebellum in language tasks. Specifically, the results supported the finding of crossed reciprocal connections between cortical higher order associations areas and the cerebellum (Hubrich-Ungureanu et al., 2002; Jansen et al., 2005; Marien et al., 2000; Mariën et al., 1996; Silveri et al., 1994). Hubrich-Ungureanu et al. (2002) showed in their fMRI study that a right-handed participant with dominant language areas located in the left neural hemisphere activated the right cerebellum in a silent verbal fluency, while a left-handed participant with right hemispheric dominant language areas activated the left cerebellum during the language task. However, a study using DTI have demonstrated that anatomical lateralization of language fiber pathways is heterogenous among individuals: approximately 60% of the cohort have left dominant connection, and remaining individuals have right dominant or symmetrical connections (Catani et al., 2007).

We found a positive correlation between LSA cosine similarity and gray matter volume in the subregion VIIIb of the right cerebellum. A study of resting-state functional connectivity revealed a strong connection between the frontoparietal network and the hemispheric VIIIb (Sang et al., 2012). Another study about cerebellar subregions and verbal working memory revealed a critical relationship between verbal working memory and gray matter density in the superior and inferior (including VIIIb) parts of the cerebellum (Cooper et al., 2012). Lastly an MRI study on the KE family members with inherited speech and language disorders, showed reduced GM volume in the posterior lobe of the cerebellum (lobule VIIIb) compared to non-affected family members and healthy controls (Watkins et al., 2002).

In summary, the evidence from multimodal approaches (neuroanatomy, clinical, fMRI, meta-analysis) on the cerebellum and nonmotor language functions show a dense network of crossed and reciprocal cerebello-cerebral connections (Marien and Borgatti, 2018).

## 5 Limitations

Our current study has several limitations. Firstly, our measure of subjective language interest is polysemantic. Language interest can include various aspects of language, writing, speaking, reading and others. Another limitation related to our measure is that we observed most of the participants rated themselves above average (Table 1). Despite the ambiguity of the question, we capture inter-individual heterogeneity of subjective interest and its association with strategy of accessing semantic space.

Other limitations are related to the LSA analysis. First, a semantic space depends on the text database included. Many of them use Wikipedia data, while others include news articles. In the current study we used the public available pretrained semantic space. We used that semantic space because it includes large number of vocabulary (526,004 words) to cover animal words in the VFT and large dimensions (400 dimensions) to detect subtle difference in a semantic space, but another semantic space derived from newspaper articles can produce slightly different results.

## 6 Conclusion

In conclusion, we could show that inter-individual heterogeneity in a strategy to access knowledge is associated with the self-perceived language interest and structural difference in the cerebellum.

## CRediT authorship contribution statement

Francilia Zengaffinen: Conceptualization, Methodology, Project administration, Investigation, Data curation, Visualization, Formal analysis, Writing - Original Draft. Stephan Furger: Project administration, Investigation. Antje Stahnke: Project administration, Investigation. Roland Wiest: Resources, Data curation. Thomas Dierks: Conceptualization, Methodology, Funding acquisition, Supervision. Werner Strik: Conceptualization, Methodology, Funding acquisition, Supervision. Yosuke Morishima: Conceptualization, Methodology, Data curation, Formal analysis, Writing - Original Draft.

## Declaration of competing interest

We, the authors, have no conflict of Interest to declare.

## Data Availability

The authors do not have permission to share data. According to the human research act in Switzerland sharing data without the explicit consent of the participant is against the law and therefore prohibited. We did not specifically ask our participants for permission and therefore cannot share this data.
