# Supplementary material for: “Computational analysis on verbal fluency reveals heterogeneity in subjective language interests and brain structure”
Source: Neuroimage Rep. 2023 Feb 19;3(1):100159. doi: 10.1016/j.ynirp.2023.100159 (PMC7615821; doi:10.1016/j.ynirp.2023.100159)
Supplement: Multimedia component 1 [file mmc1.docx]

**Supplementary material for**

**“Computational analysis on verbal fluency reveals heterogeneity in subjective language interests and brain structure”**

Francilia Zengaffinen, Antje Stahnke, Stephan Furger, Roland Wiest, Thomas Dierks, Werner Strik, Yosuke Morishima

**Supplementary Figure 1: GM volume association with age in cohort 16-40**

**Supplementary Figure 2: GM volume association with age in cohort 21-40**

**Supplementary Figure 3: GM volume association with LSA in cohort 21-40**


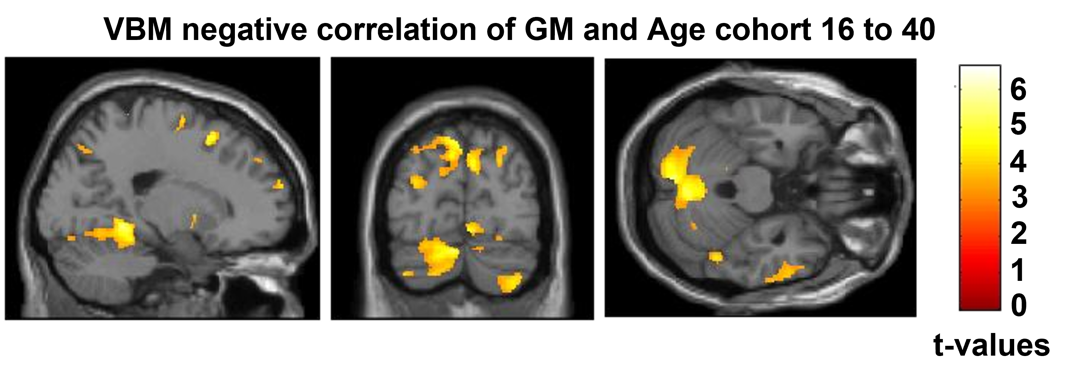


Figure S1. Statistical parametric map for negative correlation between gray matter volume and age in the cohort including the age 16 to 40. For visualization purposes, voxels that survive at p < 0.001 uncorrected (t =3.17) are depicted.


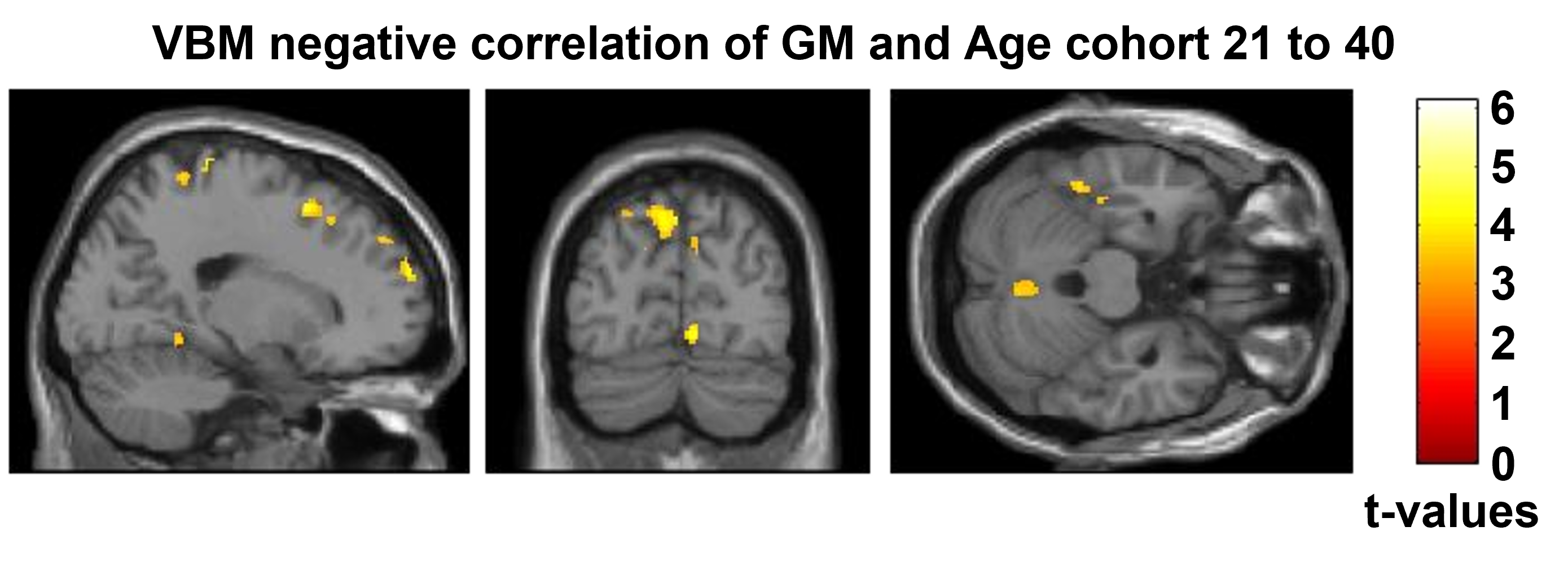


Figure S2. Statistical parametric map for negative correlation between gray matter volume and age in the cohort including the age 21 to 40. For visualization purposes, voxels that survive at p < 0.001 uncorrected (t =3.17) are depicted.


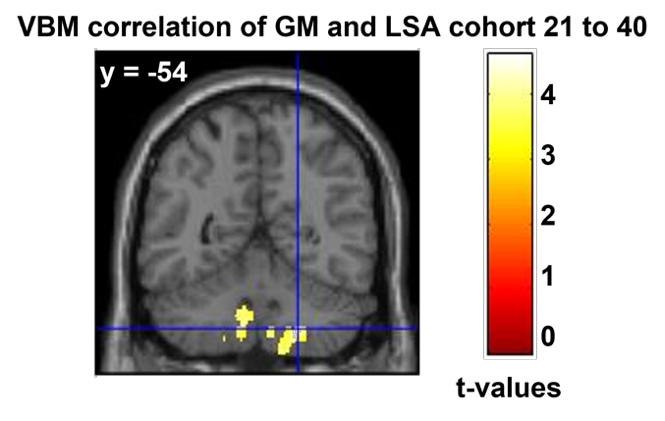


Figure S3. Statistical parametric map for correlation between LSA cosine similarity and gray matter volume in the cohort including the age 21 to 40. Right cerebellum was significantly correlated with LSA cosine similarity (peak: [x, y, z] = [21, -52.5, -48]; t-value = 4.62; p(cluster-wise FWE thresholded at p < 0.001 uncorrected) = 0.01). For visualization purposes, voxels that survive at p < 0.001 uncorrected (t =3.20, cluster size = 605) are depicted.
